# A study on the mechanism of school support to improve school adjustment of rural left-behind children——Analysis based on CEPS (2013–2014) data

**DOI:** 10.1371/journal.pone.0317459

**Published:** 2025-03-11

**Authors:** Shunxu Peng, Zhenyu Li, Jiapeng Ai, Jia Liu, Jingjing Shi, Hao Liu

**Affiliations:** 1 College of Education for the Future, Beijing Normal University, Beijing, China; 2 School of Management, Wuzhou University, Guangxi, China; 3 School of Economics, Qingdao University, Qingdao, China; 4 Collaborative Innovation Centre for Assessment of Basic Education Quality, Beijing Normal University, Beijing, China; University of Maribor, SLOVENIA

## Abstract

School adjustment is related to the educational achievements and future career development of rural left-behind children. School support is an important institutional measure to promote the school adjustment of rural left-behind children in China. Using Structural Equation Modeling (SEM) based on CEPS data, this paper analyzed the effects of school support on rural left-behind children’s school adjustment, and found that: (1) school support effectively improves rural left-behind children’s school adjustment ability; (2) school support enhances students’ school adjustment ability by improving their educational expectation and mental health, especially the effects of teachers’ relationship support and school soft environment support are significant; (3) teacher support has a greater effect on students’ school adjustment ability than environment support, and soft environment support enhances students’ school adjustment ability by improving their mental health, while the impact of hard environment support is weaker. This study provides theoretical support for the formulation of intervention policies for rural left-behind children and offers practical evidence for understanding the relationship between school education and family education.

## 1 Introduction

According to the communiqué of the Seventh National Population Census of China, the migrant population accounted for 26.04% of the country’s total population, an increase of 69.7% compared with the sixth national population census in 2010, while the rural population decreased by approximately 160 million, leading to a large number of left-behind children. According to the website of the Ministry of Civil Affairs of China, by the end of the 13th Five-Year Plan, there were 6.436 million left-behind children in rural China. And the issue of left-behind children has attracted widespread attention from society. As early as 2016, the State Council of China issued the “Opinions on Strengthening the Care and Protection of Rural Left-behind Children”, which put forward policy requirements to complete the care and service system and improve the rescue and protection mechanism. Left-behind children in rural areas face difficulties in reuniting with their parents, which prevents the full realization of familial nurturing functions [1,2]. Their health, growth confidence, learning supervision are weaker than those who are not left behind [3], and their interpersonal relationship and self-confidence [4], learning psychology, learning attitude and learning purpose [5], learning habits, learning self-efficacy, learning commitment and concentration [6] are also worse than their non-left-behind peers, especially in the level of mental health, such as anxiety, depression, loneliness, etc [7,8]. In 2023, the “Opinions on Improving the Mechanism of Collaborative Parenting in Schools, Families and Society”, which was jointly issued by the Ministry of Education of China and 13 other departments, put forward the need to strengthen school guidance on family education, with special attention to the healthy growth of children in difficult situations such as left-behind children in rural areas. How to formulate effective protective measures to mitigate the negative impacts on left-behind children in rural areas caused by the absence or inadequacy of parental care is a practical issue that policymakers need to address.

Schools are vital institutions for individual socialization, and poor school adjustment can weaken learning motivation, leading to issues like aversion to school and truancy, significantly reducing subsequent educational opportunities. As a result, the issue of school adjustment has garnered widespread attention from educators all over the world. Left-behind children spend a significant amount of time at school, having been separated from their parents for long periods. School adjustment can be considered the foundation of their school life and may even affect their future social adjustment. Ensuring the developmental rights of rural left-behind children is essential, and highly organized and professional schools can provide a certain degree of operational space to promote their school adjustment. Teacher and peer support can increase opportunities for interaction, helping left-behind children integrate into the school and class environment. This support can enhance their learning motivation and mental health, boosting their confidence in learning and life, thus compensating for the deficiencies in family education. Therefore, exploring the various factors influencing school adjustment among rural left-behind children and analyzing the relationships and underlying mechanisms among these factors can provide empirical support for designing effective intervention measures. This paper intends to explore whether school support affects the school adjustment of rural left-behind children. It analyzed the differentiated effects of various types of teacher support and environmental support on their learning and life adjustment. Furthermore, it examined how teacher support and environment support influence school adjustment through mediating pathways involving students’ self-educational expectations and mental health levels.

## 2 Literature review and research hypothesis

### 2.1 School adjustment

Cowen et al. first suggested in his AML model that school adjustment refers to students’ adaptation to changes in the learning environment and academic tasks [9]. Luckner and Pianta argued that school adjustment is the degree to which a student perceives a commitment to the school, is accepted by the other members of the school, and feels satisfied [10]. School adjustment is a multifactorial construct that includes attitudes, behaviors, cognition and social factors, involving the relationship between students and the school environment as well as their ability to adjust the environment [11,12]. It encompasses academic factors, social factors related to social and behavioral adjustment, family factors, and factors of school satisfaction [13–15]. The definition of school adjustment put forward by Ladd et al. has been widely used and it identified school adjustment as a condition in which students participate happily in school activities and achieve academic success in the school context [16]. Zou further defined school adjustment as including academic adjustment such as motivation and attitude towards learning, and non-academic adjustment including interpersonal and emotional adjustment [17]. School adjustment is gradually evolving from a focus solely on academic adjustment to a more comprehensive concept that includes both academic performance and school life. This broader definition encompasses factors such as the quality of interactions with peers and teachers, as well as overall satisfaction with the school environment. Yang et al. distinguished school adjustment into academic adjustment and life adjustment [18]. From the perspective of measuring school adjustment, Anderson et al. defined academic adjustment in terms of three dimensions: academic life style, academic performance and academic motivation [19]. Lee and Kim measured school life adjustment in terms of academic tasks, school peers, teachers, and school life [20]. Xu defined school life adjustment as learning environment adjustment, learning method adjustment, interpersonal adjustment and behavioral adjustment [21]. School maladjustment can lead to problems such as dropping out of school, reduced motivation to learn, and truancy, and can also affect later educational achievement and career development. Those students who are well adjusted in school are less likely to be involved in bullying than maladjusted students [22]. Research on school adjustment among left-behind children in China is relatively abundant, primarily focusing on the impact of parental migration on these children, including studies on learning, mental health, and social behavior. However, there is still a lack of research examining school adjustment specifically from the school’s perspective. Yao and Mao found that mother migration had the greatest impact on the academic performance of left-behind children in rural China [23]. By examining the impact of rural parental mobility on the school behavior of older rural left-behind children, Song found that parental mobility had a negative effect on left-behind children’s class integration and behavioral norms [24]. Ning and Zhou found that non-left-behind children were better than left-behind children on all dimensions of school adjustment, and that family closeness and adaptability had a significant positive predictive effect on rural left-behind children’s school adjustment [25].

### 2.2 Teacher support

Teachers play an important role in improving students’ school adjustment. As an important part of the social support system, teacher support refers to teachers’ expression of concern, listening, understanding, affection and encouragement for students [26]. Reddy et al. found that students who perceived increased teacher support had fewer depressive symptoms and higher self-esteem [27]. Teacher support promotes children’s school adjustment [28,29], and support from teachers and peers makes adolescents feel satisfied with school and education, and may increase their chances for healthy development [30].

Research has confirmed that perceived teacher support positively predicts secondary school students’ engagement in learning [31], that teachers providing academic support can help students reduce involvement in distractive and deviant behaviors [32] and increase students’ self-confidence in learning, which can positively predict students’ academic achievement [33], and that social support from teachers reduces students’ health-risk behaviors [34].

Pianta and Allen stated that positive relationships between students and adults (teacher-student relationships) during secondary school may be the most important factor contributing to healthy adolescent development [35]. Good teacher-student relationships contribute to students’ school adjustment [32] and are associated with increased students’ school engagement and achievement [36]. Negative teacher-student interactions, on the other hand, may allow students to develop negative self-cognition and sense of incapacity, negative attitudes towards school and authority, negative perceptions of peers and rejecting behaviors, increase the probability of antisocial behaviors, and decrease academic performance [37].

Students perceive that teachers’ emotional support significantly reduces the occurrence of academic burnout [38]. Wu et al. found that teachers’ emotional support increased children’s willingness to make friends thereby enhancing their agreeableness and extraversion, which particularly helped to improve the social skills and pro-social behaviors of children from disadvantaged groups (e.g., rural left-behind and migrant children) [39]. Teachers’ positive emotional expression is a significant predictor of students’ socio-emotional competence and school adjustment, while the interaction between teachers’ emotional expression and teachers’ negative emotional responses to students is a significant predictor of students’ socio-emotional competence and school adjustment [40]. Therefore, it is evident that teacher support for students is multifaceted, including provide academic guidance, engage in meaningful conversations with students to help alleviate their psychological concerns, and establish positive relationships by being a friend to students. And the impact of this support on students is also diverse. As a result, this paper proposes Hypothesis 1: All three dimensions of teacher support (academic support, emotional support, and relationship support) have a significant effect on school adjustment (academic adjustment and life adjustment) of left-behind children in rural areas.

### 2.3 Environment support

Educational environment refers to the specific occasions where educators and educated people live together, implement education and exert influence. The environment itself is also an educational factor that plays a subtle role in human beings [41]. Ecosystem theory emphasizes that individual development is the result of interaction between the individual and the environmental system in which he or she lives [42]. Individual-environment interaction theory suggests that human cognitive behaviors are influenced by the environment, while at the same time the individual actively and proactively acts on the environment, and that individual behaviors are influenced by a combination of dispositional tendency and external environmental factors [43]. Therefore, students’ school adjustment depends not only on support from teachers, peers, and parents, but is also influenced by their own interactions with the environment in which they live, whether it is the hard environment formed by physical facilities, equipment, etc., or the soft environment formed by non-physical systems, management, etc., which probably have a significant impact on students. The physical environment serves as the material foundation for the functioning of a school, while the non-physical environment directly determines the extent to which the school can operate effectively. Together, they form the environmental factors that influence student development. The United Nations International Children’s Emergency Fund proposed in 2000 to create child-friendly schools in terms of both physical conditions and psychological support, so that children’s creativity can be developed in a quality educational environment [44]. Some scholars have suggested that the school environment includes organization, teaching and learning, interpersonal relationships, culture and values [45]. Li et al. categorized school environment into physical environment, teacher-student relationship, peer relationship, classroom climate and regulations [46]. Kou and Wang distinguished the school environment into hard and soft environments, the hard environment refers to the objective environment of the campus and classroom and the arrangement of activities, and the soft environment mainly refers to the moral environment, social opinion and evaluation system, they found that the environment can constrain the students’ undesirable behaviors and promote the occurrence of pro-social behaviors [47]. Yin et al. distinguished between hard and soft environments and found that the school environment enhances the positive emotions and psychological states of left-behind children and significantly increases their pro-social behaviors by improving children’s psychological capital and social satisfaction [48]. Hou and Zhao found that school hardware facilities, human resources, and management level had a positive effect on the school adjustment of migrant children [49], and Moos was the first to propose and use the term “school climate” to assess the educational and teaching environment [50]. Zhao et al. found that school climate affects achievement motivation of left-behind children in rural China through academic adjustment, which is moderated by teacher support [51]. Class is an integral part of school and those students who perceived a cohesive and competitive class environment scored higher on school liking, interest in learning, academic efficacy, and self-confidence than average and conflict students [52]. Yang and Dai pointed out that students’ disciplinary activities and teachers’ educational styles are micro-representations of the inner school climate, which directly affects adolescents’ psycho-emotional well-being, and confirmed a positive correlation between inner school climate and adolescents’ psychological well-being by defining the school climate as the activities of delinquent youths in the vicinity of the school [53]. Du and Zhang defined school environment as the type of school, peer environment and interpersonal relationships, and found that the school environment had a significant positive effect on the resilience of left-behind children, and that the school environment also promotes the role of the family environment [54]. Although different researchers define the school environment differently based on their research objectives, particularly regarding the concept of the soft school environment, but there is a consensus that both the hard and soft environments significantly impact student development, which leads to our Hypothesis 2: School environmental support (both hard and soft) has a significant effect on the school adjustment of rural left-behind children.

### 2.4 The intermediary mechanism of school support affecting students’ school adjustment

Mental health is a manifestation of an individual’s good mental quality and a sustained psychological state in which the individual has vitality of life, positive inner experience, good social adjustment, and is able to effectively utilize the individual’s physical and mental potentials and positive social functions [55]. Left-behind children face more psychological problems due to the lack of parental support and are significantly less likely than non-left-behind children in terms of interpersonal relationships and self-confidence [4]. Based on latent profile analysis, Zhao et al. found that students in the high mental quality group had the best school adjustment status, followed by the medium mental quality group, and the poorest school adjustment was among students in the low mental quality group [56]. Xiong et al. found that the teacher-student relationship affects left-behind children’s school adjustment through psychological health and learning engagement, and that psychological health and learning engagement play a chain mediating role between the teacher-student relationship and school adjustment of left-behind children [57]. Yang et al. explored the effects of teacher support on migrant children’s school adjustment in terms of teachers’ academic support, emotional support, and relationship support, and pointed out that teacher support played a partially mediating role by improving migrant children’s self-educational expectation and mental health status [18]. Chen et al. classified teacher support into learning support, ability support, and emotional support, and found that mental quality played a fully mediating role in the overall teacher support, learning support, and ability support affecting academic achievement, and played a partially mediating role in the relationship between teachers’ emotional support and academic achievement [58]. It can be inferred that mental health levels may serve as a mediating variable in the relationship between teacher support and student adjustment.

Self-educational expectation is an important mediating variable used by the Wisconsin model to explain the influence of family socioeconomic status on educational achievement [59]. Yang verified that parental engagement influenced students’ school adjustment through students’ self-educational expectation [60]. It has been noted that schools of different quality influence individuals to form different levels of self-educational expectation [61]. It can be inferred that students’ self-educational expectation may also serve as a mediating variable in the influence of factors such as family support, school environment, and teacher support on students’ school adjustment.

**Fig 1:**
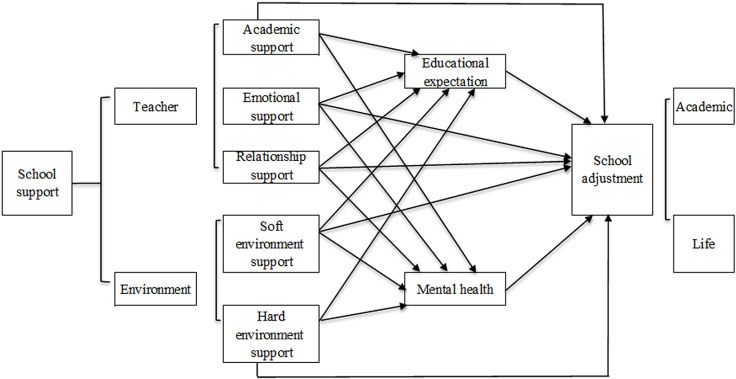
Schematic diagram of the impact mechanism of school support on promoting school adjustment of left-behind children.

Based on the above two inferences, this paper proposes Hypothesis 3: School support contributes to rural left-behind children’s school adjustment by influencing their educational expectation and mental health. By incorporating teachers and the environment into the unified analytical framework of school support, a schematic diagram of school support influencing rural left-behind children’s school adjustment was established as shown in Fig 1.

## 3 Materials and methods

### 3.1 Data sources

The data used in this paper come from the China Educational Panel Survey (CEPS) implemented by the National Survey Research Center at Renmin University of China, which conducted a random survey of 28 county-level units in China, using the 2013–2014 school year as the baseline, with the average level of education of the population and the proportion of the migrant population as the stratification variables, and adopting the multi-stage probability proportional to size sampling methodology, and sampling a total of 112 schools with 438 classes. The survey included basic information about students and parents, family environment, parent-child interactions, physical and mental health, educational expectation, parent-teacher interactions, and student-teacher relationships, peer relationships, etc. The first phase of the survey involved 10,279 seventh-grade junior high school students and 9,208 junior high school ninth-grade students. The student questionnaires were matched with parent questionnaires, and a total of 2,833 rural left-behind children were screened according to student household registration types and whether or not their parents were living at home with them (samples in which only one parent was at home or neither parent was at home were retained), and a total of 7,854 rural non-left-behind children (who were living with both parents) were also used as a control sample. In addition, when using regression and structural equation modelling analysis, it was specified that the person completing parental questionnaire should be neither the mother or the father. The sample size of left-behind children in rural areas is 1,874, while the sample size of non-left-behind children in rural areas is 7,453.

As the CEPS is a nationally representative survey approved and conducted by a reputable academic institution, it is designed and implemented with the highest standards of research ethics, ensuring participant confidentiality, and adhering to all relevant guidelines. Since the data are collected at the national level and do not involve direct interaction with individual participants, no specific ethical approval from an ethics committee was required.

### 3.2 Variables Introduction

1. The dependent variable is school adjustment. The school adjustment that this study focuses on includes two aspects, students’ adjustment to school academy and students’ adjustment to school life. Academic adjustment reflects the performance of left-behind children in school, with academic performance serving as a proxy variable for academic adjustment. Regarding school academic performance, the CEPS student questionnaire asked whether students currently find it difficult to learn Math, Chinese, and English, and assigned “particularly difficult,” “somewhat difficult,” “not very difficult,” and “not difficult at all.” to values of 1, 2, 3, 4, and then sum up these three items to get the student’s academic adjustment score. The larger the value, the stronger the student’s academic adjustment. Life adjustment reflects the school life of left-behind children. Regarding school life, eleven questions in the CEPS student questionnaire is selected, and six questions (such as “Most classmates in my class are friendly to me” and “I find it easy to get along with others”) are positively framed measurements, asking about school life and each question is assigned a value of 1, 2, 3, and 4 respectively based on the responses of “completely disagree,” “somewhat disagree,” “somewhat agree,” and “completely agree.” And, five negatively framed questions (“I am often late”, “I often skip classes”, “My parents often receive criticism from teachers”, “I feel bored in this school” and “I hope to go to another school”) are scored in reverse and summed with the six positive questions to get the student’s school life adjustment score. The larger the value, the stronger the student’s school life adjustment ability.
2. The independent variable is school support. Previous research paid more attention to teacher support. This study not only focused on teacher support but also included school environment support into school support system, further broadening the research content. Teacher support is divided into three dimensions, academic support, emotional support and relationship support. In the CEPS student questionnaire, respondents were asked whether they agreed that Math, Chinese and English teachers often asked themselves academic questions, and assigned the values 1, 2, 3, 4 to “completely disagree”, “somewhat disagree”, “somewhat agree” and “completely agree” respectively, and summed to obtain the score of teachers’ academic support for students. The larger the value, the higher the academic support of teachers. Emotional support uses the CEPS student questionnaire items which asking respondents whether their Math, Chinese, English and head teachers often praise them, and assign “completely disagree”, “relatively disagree”, “somewhat agree” and “completely agree” as 1, 2, 3, 4 respectively, and summed to obtain the score of teachers’ emotional support for students. The larger the value, the greater the emotional support teachers provide to students. Teachers’ relationship support uses four questions from the parent questionnaire asking whether the teachers are responsible and patient with the child, and whether the child likes the head teacher and other teachers. The teachers’ responsibility and patience were assigned “not at all” 1, “not too much” 2, “normally” 3, “fairly” 4, and “very” 5 respectively, while whether the child likes the head teacher and other teachers were assigned “not at all” 1, “not too much” 2, “fairly” 3, “very” 4, and then add up the four questions to get the teachers’ relationship support score. The larger the value, the greater the teacher relationship support. In addition, school environment support includes school soft environment support and school hard environment support. The soft environment support in the school uses the school leaders’ questionnaire to get descriptions of the school’s situations about “students often playing truant” and “school discipline is difficult to manage”, as well as incidents such as “students fighting, vandalizing public property, smoking and drinking, participating in gang activities, and disordered classroom discipline” last week, respectively. Assign values 1, 2, 3, 4 to “completely consistent”, “somewhat consistent”, “not consistent” and “not consistent at all”, and according to the frequency of occurrence, “more than ten times”, “five to ten times”, “one to four times” “Never happened” were assigned values of 0, 1, 2, and 3 respectively. We also asked the head teacher about the school’s management of students, and assigned values of 1, 2, 3, 4 and 5 respectively according to “very loose”, “relatively loose”, “generally”, “relatively strict”, and “very strict”, and then add up these 9 items to get the school soft environment support score. The larger the value, the better the school soft environment, which also means the higher the school management quality. The school hard environment mainly refers to the hardware facilities within the school, using the school leader’s questionnaire answered whether the school has a circular track, related venues (including laboratories, computer classrooms, libraries, student activity rooms, psychological counseling rooms, student restaurants, and sports fields) and whether “all class access” is achieved. The circular track is based on “No, yes” and are assigned values of 0,1 respectively, venues are assigned values of 0, 1, 2 respectively according to “no”, “yes, but the equipment needs to be improved”, “yes, and the equipment is good”, “all class access” is assigned the values of “not at all”, “some classrooms”, “most classrooms”, and “all classrooms” are assigned values of 0, 1, 2, and 3 respectively, and then these 12 items are summed to obtain the school’s hard environment score. The larger the value, the greater the support of the school’s hard environment.
3. The mediating variables were students’ self-educational expectation and mental health. The CEPS student questionnaire asked respondents “what level do you want to be educated”, and the answers were converted into years according to the degree of the answer from the low to the high, and the higher the value indicated the higher the self-educational expectation. For mental health status, the student questionnaire asked the respondents whether they had been frustrated, depressed, unhappy, uninterested in life, sad, etc. in the past seven days, and converted the answers from “always”, “often”, “sometimes”, “seldom”, “never” to assigned values 1, 2, 3, 4, 5, and these five questions were summed up to get a measure of the students’ mental health status, and the larger the value means the higher the mental health of the students.
4. Control variables include demographic and family background variables. The demographic variables include gender (female =  0, male =  1), only child (only child =  0, non-only child =  1), and grade level (grade 7 =  0, grade 9 =  1); the family background variables include the parents’ education level, the family’s economic condition (poor = 1, moderate = 2, rich = 3), parental relationship, and parent-child relationship. With regard to parental relationship, a student questionnaire asking students about their parents’ quarrels and relationship was used, with “My parents quarrel a lot”, “It is not like that” and “It is like that” reverse scored 2 and 1, and “My parents have good relationships with each other”, “It is not like that” and “It is like this” were scored 1 and 2, and these two items were summed to obtain a measure of parental relationship, with the larger the value the better the parental relationship. The parent-child relationship was measured by asking students how they related to their parents, assigning a value of 1, 2, 3 to their relationship with their mothers and fathers according to the categories of “not close,” “generally”, “very close,” and summing these two items to obtain the parent-child relationship variable, the larger the value of which implies the better parent-child relationship.

### 3.3 Models

#### 3.3.1 OLS.

In order to test the effect of school support on school adjustment of rural left-behind children and the possible pathways that exist for school support to influence school adjustment by enhancing students’ educational expectation and mental health (as proposed in Hypothesis 3), the present study firstly used general linear regression and developed the following regression model:


$$Y=\beta_{0}+\beta_{1}X_{1}+\beta_{2}X_{2}+\beta_{3}X_{3}+\beta_{4}X_{4}+\beta_{5}Z_{5}+\beta_{6}Z_{6}+\beta_{7}Z_{7}+\overset{14}{\underset{k=8}{\sum }}\beta_{k}X_{k}+\varepsilon$$ (1)

where, Y denotes students’ school adjustment, X_1_, X_2_, X_3_, X_4_, X_5_ denote teachers’ academic support, teachers’ emotional support, teachers’ relationship support, school soft environment support, and school hard environment support, Z_6_, Z_7_ denote students’ educational expectation and mental health, respectively, and X_k_ (k =  8–14) denote grade, gender, parental relationship, parent-child relationship, parents’ education level, only child, and family economic condition, respectively, and *β*_*i*_ is the parameter to be estimated. *β*_*0*_ is the constant term, and *ε* is the error term. The core explanatory variables are teacher support and environment support.

#### 3.3.2 SEM.

**Fig 2:**
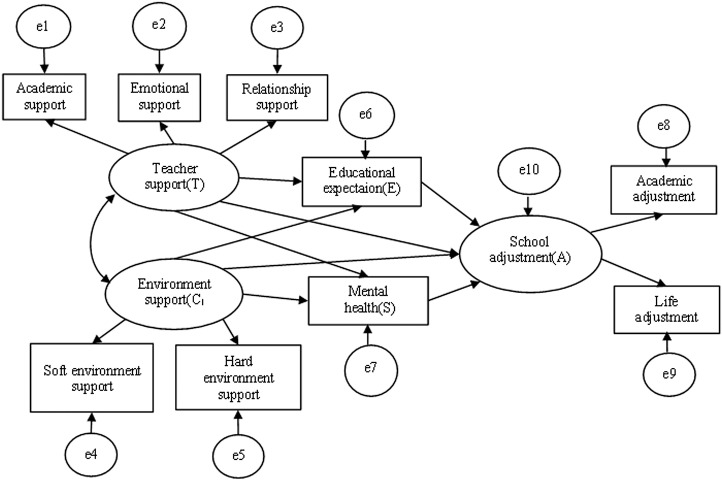
Structural equation model of school support affecting students’ school adjustment.

Next, a structural equation model is used to further validate the mediating mechanism proposed in Hypothesis 3. Structural equation model (SEM) is an extension of the general linear model and has advantages over linear regression models in that it can handle multiple dependent variables simultaneously, allows independent and dependent variables to be latent variables, can contain measurement error, latent variables can be measured using multiple indicators, and an indicator variable can be subordinate to different latent variables. In order to test the hypotheses proposed in the previous section, the following structural equation model was set up, as shown in Fig 2. Teacher support (T), environment support (C), and school adjustment (A) were all latent variables, students’ educational expectation (E) and students’ mental health (S) served as observable variables as well as mediators, and *e*_*k*_ (k = 1–10) was the error term, and teacher support and environment support were set to have a reciprocal relationship. To ensure that the model would work in Amos, mean interpolation was used to add missing values for each variable.

## 4. Results

### 4.1 Comparative analysis of the differences between left-behind and non-left-behind children in rural areas

As shown in Table 1, there are significant differences between rural left-behind children and non-left-behind children in many aspects. Except for no significant difference between left-behind children and non-left-behind children in terms of educational expectation, left-behind children are lower than their non-left-behind counterparts in terms of learning adjustment, life adjustment, teacher support, environment support, and mental health, which highlights the disadvantaged position of left-behind children in rural China. Besides, the proportion of boys and non-only children among left-behind children is higher than that of non-left-behind children, and their family economic backgrounds are also relatively weak; for example, the highest level of education of left-behind children’s parents is about 0.29 years lower than that of non-left-behind children’s parents on average, and their family economic conditions, parental relationships and parent-child relationships are significantly worse than those of non-left-behind families. Improving left-behind children’s school adjustment ability by upgrading the level of school support will help rural left-behind children achieve better development through school education.

**Table 1:** Comparison of differences between rural left-behind children and non-left-behind children.

		Full samples	Left-behind children	Non-left-behind children	Difference comparison
Dependent variables	School adjustment				
	Academic adjustment	7.097	6.918	7.142	−0.224***
	Life adjustment	33.714	32.941	33.906	−0.965***
Independent variables	Teacher support				
	Academic support	8.051	7.823	8.107	−0.284***
	Emotional support	9.446	9.073	9.539	−0.467***
	Relationship support	14.937	14.580	15.026	−0.446***
	Environment support				
	Soft environment support	25.048	24.703	25.175	−0.472***
	Hard environment support	13.915	12.943	14.283	−1.340***
Mediator	Students’ educational expectation	16.060	15.970	16.083	−0.113
	Students’ mental health	19.594	19.056	19.728	−0.673***
Control variables	Gender	0.517	0.547	0.510	0.038***
	Only child	0.725	0.761	0.716	0.045***
	Grade	0.483	0.478	0.484	−0.007
	Parental education level	9.539	9.305	9.598	−0.292***
	Family economic conditions	1.773	1.682	1.796	−0.113***
	Parental relationship	3.751	3.633	3.780	−0.148***
	Parent-child relationship	3.292	3.098	3.339	−0.241***

Note: * , **, and *** indicate significance at the 10%, 5%, and 1% significance levels respectively.

### 4.2 Analysis of the mediating effect of school support on students’ school adjustment

Table 2 preliminarily explores the relationships between the dimensions of school adjustment, school support, and the mediating variables, and the results show that, except for teachers’ academic support and emotional support, which are not correlated with the school’s hard environment support, educational expectation, which are not correlated with the school’s soft environment support and hard environment support, and mental health, which is not correlated with the school’s hard environment support and educational expectation, the dimensions of school adjustment, school support, educational expectation, and mental health of the left-behind children in rural China are all correlated with each other, which to some extent fulfils the prerequisites for conducting mediation analysis.

**Table 2:** Correlation matrix of main variables of rural left-behind children.

Variables	1	2	3	4	5	6	7	8	9
1 Academic adjustment	1								
2 Life adjustment	0.30***	1							
3 academic support	0.23***	0.26***	1						
4 emotional support	0.22***	0.32***	0.64***	1					
5 Relationship support	0.25***	0.35***	0.31***	0.29***	1				
6 Soft environment support	0.10***	0.14***	0.11***	0.09***	0.12***	1			
7 Hard environment support	0.09***	0.09***	0.01	0.01	0.08***	0.12***	1		
8 Educational expectation	0.26***	0.19***	0.17***	0.14***	0.16***	−0.02	−0.00	1	
9 Mental health	0.18***	0.39***	0.15***	0.20***	0.22***	0.07***	0.05	0.05	1

Table 3 presents the results of the effect of school support on school adjustment of rural left-behind children. Specifically, Model 1 did not control for any variables, and teachers’ academic support, emotional support, relationship support, and school environment (soft and hard environment) support all significantly increased students’ school adjustment. After controlling for relevant variables, Model 2 results were consistent with Model 1 results. Hypothesis 1 was verified. Model 3 showed that teachers’ emotional support, relationship support, and school environmental (soft and hard environment) support all significantly improved students’ school life adjustment, and the effect of teachers’ academic support on students’ life adjustment was positive but not significant. After controlling for the relevant variables, the results in Model 4 were consistent with Model 3 except that there is no significant effect of teachers’ academic support on students’ school life adjustment. As shown by the results of Model 2 and Model 4, the research Hypothesis 1 was initially verified. The results of Model 5 show that teachers’ academic support, emotional support and relationship support significantly increase students’ educational expectation, while school soft environment support significantly decreases students’ educational expectation, and school hard environment support has no significant effect on students’ educational expectation. Even after controlling for relevant variables, the results of Model 6 remain consistent with Model 5. Model 7 showed that teachers’ emotional support, relationship support, and school soft environment support all significantly increased students’ mental health, while teachers’ academic support and school hard environment support had no significant effect on students’ mental health. After controlling for relevant variables, the results of Model 8 are basically consistent with Model 7. The results of Model 9 show that only the mediating effects of teachers’ academic support, relationship support, and school soft environment support are significantly present, and the mediating effects of teachers’ emotional support and school hard environment support on students’ academic adjustment are not significant. Model 11 shows that only the mediating effects of teachers’ academic support on students’ school life adjustment are not significant. After controlling for relevant variables, Model 10 and Model 12 remained consistent with the results of Model 9 and Model 11.

**Table 3:** Results of the impact of school support on school adjustment of rural left-behind children.

	Model 1	Model 2	Model 3	Model 4	Model 5	Model 6	Model 7	Model 8	Model 9	Model 10	Model 11	Model 12
	Academic adjustment	Academic adjustment	Life adjustment	Life adjustment	Students’ educational expectation	Students’ educational expectation	Students’ mental health	Students’ mental health	Academic adjustment	Academic adjustment	Life adjustment	Life adjustment
Teachers’ academic support	0.089***	0.074***	0.059	0.037	0.153***	0.158***	0.001	0.014	0.063 *	0.052	0.065	0.043
Teachers’ emotional support	0.061***	0.047**	0.329***	0.305***	0.073 *	0.065 *	0.184***	0.143***	0.038	0.031	0.235***	0.232***
Teachers’ relationship support	0.136***	0.112***	0.478***	0.448***	0.153***	0.136***	0.250***	0.185***	0.118***	0.094***	0.351***	0.352***
School soft environment support	0.044***	0.031**	0.117***	0.106***	−0.062**	−0.084***	0.071**	0.071**	0.052***	0.039**	0.127***	0.114***
School hard environment support	0.030***	0.025**	0.053**	0.058**	0.003	0.001	0.032	0.041 *	0.022	0.017	0.044 *	0.047 *
Students’ educational expectation									0.115***	0.104***	0.164***	0.155***
Students’ mental health									0.052***	0.053***	0.320***	0.309***
Grade 9		−0.363***		0.792***		−0.123		−0.004		−0.367***		0.855***
Male		−0.423***		−0.670***		−0.460**		0.731***		−0.365***		−0.701***
Parental relationship		−0.009		0.510**		0.043		0.679***		−0.065		0.285 *
Parent-child relationship		0.163***		0.590***		0.207**		0.545***		0.113 *		0.393***
Parental education level		0.101***		0.100**		0.117***		0.059		0.084***		0.063
Non-only child		0.148		−0.476 *		0.132		−0.101		0.088		−0.567**
Generally		0.165		−0.153		−0.274		−0.085		0.233 *		−0.116
Rich		0.588 *		−1.784***		0.083		−0.748		0.765 *		−1.561**
Constant term	2.246***	1.951***	18.911***	15.858***	13.432***	12.777***	11.527***	7.700***	−0.028	0.266	12.809***	11.259***
Adjusted R^2^	0.106	0.143	0.193	0.232	0.042	0.057	0.069	0.115	0.159	0.184	0.276	0.299
N	1587	1486	1503	1416	1532	1434	1547	1456	1462	1377	1389	1314

Note: (1) * , **, and *** indicate significance at 10%, 5%, and 1% significance levels respectively. (2) The standard error of the coefficient is omitted. (3) Models 1 to 12 all use seventh grade, girls, only children, and difficulties as the benchmark group.

Model 10 shows that the effects of teachers’ academic support and emotional support on students’ academic adjustment are fully mediated through the mediating effects of students’ educational expectation and mental health, and the effects of school soft environment support on students’ academic adjustment are partially mediated through the mediating effects of students’ educational expectation and mental health. The mediating effect of school hard environment support on students’ academic adjustment was not significant. Compared to Grade 7, Grade 9 students’ academic adjustment was significantly lower, and boys were less academically adjusted than girls, parent-child relationships had a positive effect on students’ academic adjustment, more educated parents improved students’ academic adjustment, and children with better economic conditions were significantly more academically adjusted. Model 12 shows that the effect of teachers’ academic support on students’ life adjustment is fully realized through students’ educational expectation and mental health, while teachers’ emotional support, relationship support, as well as support for the soft and hard environments within the school are partly realized through students’ educational expectation and mental health. Grade 9 students were more resilient than Grade 7 students, boys were less resilient than girls, better parental relationships were associated with greater school adjustment, parent-child relationships were significant in enhancing students’ school adjustment, non-only children were significantly less resilient than only children, and children from wealthy families were significantly less resilient than children from struggling families. Combining Model 10 and Model 12, Hypothesis 3 was verified and Hypothesis 2 was partially verified. However, it remains unclear whether the effects of teacher support and environment support on students’ academic and life adjustment are mediated through students’ educational expectation or mental health, or both.

**Fig 3:**
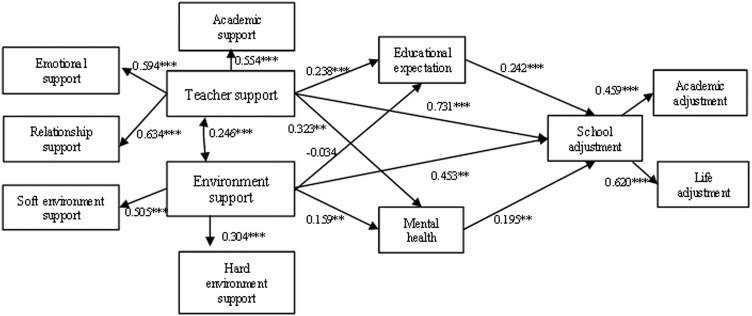
Standardized coefficient chart of school support in promoting school adjustment of left-behind children.

Therefore, according to the constructed structural equation model (e.g., Fig 2), the occurrence mechanism of school support influencing students’ school adjustment was further explored. Firstly, the fitness test of the original model and data was conducted, the chi-square degrees of freedom ratio of the original model was CMIN/DF =  9.830 and RMSEA =  0.069, which indicated that the fitness of the model to the data was not up to the standard, and after correcting the original model, the chi-square degrees of freedom ratio of the final model was reduced to CMIN/DF =  3.694, the root-mean-square-of-error (RMSEA) =  0.038 <  0.05, and the rest of the metrics such as AGFI =  0.980, GFI =  0.995, TLI =  0.959, CFI =  0.986, and NFI =  0.982 are all greater than 0.9, which can be considered as a good model fit since the sample size used is more than 1800. The results of the model estimation are shown in Fig 3. Specifically, teacher support and environment support have a direct and significant effect on rural left-behind children’s school adjustment and teacher support influences students’ school adjustment through two paths: students’ educational expectation and mental health, with students’ educational expectation and mental health showing a significant mediating effect. School environment support had a negative but not significant effect on students’ educational expectation, while it had a significant positive effect on students’ mental health. The results of the structural equation analyses reaffirmed Hypotheses 1 and 2, and partially verified Hypothesis 3. It was supposed that the effect of environment support on school adjustment may not be mediated through the pathways of students’ educational expectation and/or students’ mental health.

The bias-corrected nonparametric estimation method in the bootstrap procedure was used to repeat the sampling 2000 times and calculate its 95% confidence interval. The mediation effect was considered significant if the bootstrap 95% confidence interval did not contain zero. The difference between the impact effects of the two paths was also tested. The estimation results of the impact effects of specific paths are shown in Table 4. Specifically, the total effect of T-E-A was significant (95% confidence interval [0.488, 1.977], p =  0.001), while the indirect effect of T-E-A was significant (95% confidence interval [0.320, 1.049], p =  0.001), and the mediating effect accounted for 58.2% of the total effect (0.681 ÷  1.170), which suggests that students’ educational expectation play a considerable role in teacher support affecting students’ school adjustment. From the T-S-A, the total effect was also significant (95% confidence interval [0.614, 2.574], p =  0.001), as well as the indirect effect (95% confidence interval [0.437, 1.617], p =  0.001), and the mediating effect accounted for 66.6% (0.975/1.464), which suggests that teacher support also plays a considerable role by improving students’ mental health facilitated students’ school adjustment. Part of Hypotheses 1 and 3 were again verified. Comparing the effects of the two paths revealed that the effect of T-E-A was weaker than that of T-S-A and that there was a significant difference in this influence effect.

**Table 4:** Comparison of effects of different influencing paths.

Effect	Path	Estimated value	Lower bound of 95% confidence interval	Upper bound of 95% confidence interval	p value
Indirect effect	T-E-A	0.681	0.320	1.049	0.001
	T-S-A	0.975	0.437	1.617	0.001
Direct effect	T-A	0.488	0.170	0.995	0.001
total effect	T-E-A	1.170	0.488	1.977	0.001
	T-S-A	1.464	0.614	2.574	0.001
Difference		−0.294	−0.796	−0.043	0.019
indirect effect	C-E-A	−0.041	−0.368	0.240	0.751
	C-S-A	0.548	0.196	1.129	0.004
Direct effect	C-A	0.331	0.174	0.705	0.001
Total effect	C-E-A	0.290	−0.128	0.806	0.152
	C-S-A	0.878	0.426	1.859	0.001
Difference		−0.589	−1.134	−0.199	0.002
Total effect	T-E-A	2.145	0.912	3.543	0.001
	T-S-A				
	C-E-A	0.837	0.184	1.934	0.019
	C-S-A				
Difference		1.308	−0.036	2.959	0.058
Total effect	T-E-A	2.982	1.679	4.883	0.001
	T-S-A				
	C-E-A				
	C-S-A				

In terms of the two paths, C-E-A and C-S-A, the indirect effect of C-E-A is negative and insignificant, and the indirect effect of C-S-A is significantly positive, indicating that the mediating effect of C influencing A through influencing E and then A does not exist, but the direct effect of C-A is significant (p =  0.004). Overall, the path of C influencing A is mainly through C influencing S and thus A, rather than through influencing E. The test results also show that the path effect of C-E-A is significantly lower than that of C-S-A (95% confidence interval [−1.134, −0.199], p =  0.002). This result echoes the results of Model 5 to Model 8 in Table 3, in which soft environment support in school has a significant negative effect on students’ educational expectation and a significant positive effect on students’ mental health, while the effects of hard environment support in school on students’ educational expectation and mental health are largely insignificant, from which it is judged that environment support embodies mainly the effects of the soft environment in school, and that it does so through influencing students’ mental health. It is judged that environment support mainly reflects the effect of soft environment in school, and it is through affecting students’ mental health that it affects students’ school adjustment.

Overall, school support positively contributes to rural left-behind children’s school adjustment with an effect of 2.982 (95% confidence interval [1.679,4.883], p =  0.001), the effect of influencing students’ school adjustment (A) through teacher support (T) is 2.145 (95% confidence interval [0.912,3.543], p =  0.001), and the effect of influencing school adjustment (A) through environment support (C) was 0.837 (95% confidence interval [0.184,1.934], p =  0.019), which numerically indicates that the T-A effect is approximately 2.5 times greater than the C-A effect. This shows that both teacher and environment support play a role in school support influencing students’ school adjustment, but teacher support plays a significantly stronger role than environment support.

## 5 Discussion

### 5.1 Further discussion on the mechanisms of teacher support and environment support

There are still doubts about the influence of teacher support and environment support on rural left-behind children’s school adjustment by increasing educational expectation and mental health, one of which is that Model 5 and Model 6 in Table 3 show that the effect of school soft environment support on students’ educational expectation is negative. In order to further verify the mechanism by which the mediating effect occurs, teacher support and environment support were regressed on students’ educational expectation and mental health, respectively. The results are shown in Table 5. Model 13 to Model 16 show that teacher support has a positive and significant effect on both students’ academic adjustment and life adjustment (teachers’ academic support has a non-significant effect on life adjustment in Model 16), while Model 25 to Model 28 shows that environment support also has a positive and significant effect on both students’ academic adjustment and life adjustment. However, the mechanisms by which teacher support and environment support affect students’ academic adjustment and life adjustment are not consistent. Specifically, as shown in Model 17 and Model 18, teachers’ academic support and teachers’ relationship support significantly increase students’ educational expectation, and teachers’ emotional support has a limited effect on students’ educational expectation. And, as shown in Model 29 and Model 30, neither school soft environment support nor school hard environment support increases students’ educational expectation, and the results of Model 30 show that soft environment support in school even significantly reduces students’ educational expectation. In response to this result, it was found that the most important factor influencing students’ educational expectation is the level of parental education, followed by parent-child relationship (Due to space limitations, the specific testing process is not reported). This result is similar to the findings of Wei and Ma [62]. Students’ educational expectations are influenced by external factors from the social structure shaped by societal rules and the education system, as well as internal factors stemming from self-preference and self-efficacy [63]. Left-behind children in rural areas, situated in a low social class and facing fragmented family environments, often develop a lack of confidence or even feelings of inferiority and sensitivity. Their educational expectations tend to be relatively stable [64], and a positive school environment may not only fail to provide positive motivation but could also lead them to “withdraw,” thereby lowering their self-educational expectations. From Model 19 and Model 20, teachers’ emotional support and teachers’ relationship support can significantly enhance students’ mental health, and another test found that parent-child relationship is also an important factor in enhancing students’ mental health. Nevertheless, from the results of Model 31 and Model 32, the soft environment support in school has a significant effect on students’ mental health. Based on this result, it is again verified that teacher support, especially teachers’ relationship support, has a very important effect on students’ school adjustment, specifically, teachers’ academic support mainly affects students’ academic adjustment, teachers’ emotional support mainly affects students’ life adjustment, teachers’ academic support affects students’ academic adjustment by raising students’ educational expectation, and teachers’ emotional support affects students’ mental health by improving students’ life adjustment, teachers’ relationship support affects students’ academic adjustment and life adjustment through both educational expectation and mental health, and school soft environment support affects students’ academic adjustment and life adjustment by affecting mental health. In this study, the influence mechanism of teacher support on rural left-behind children aligns with the findings of Yang et al regarding the impact of teacher support on the school adjustment of migrant children [18]. This consistency indicates a certain stability in the effect of teacher support on student adjustment across different student groups. The supportive environment created by the school atmosphere can meet students’ needs for autonomy, interpersonal relationships, and skill development, fostering a positive learning attitude, enhancing their academic engagement and performance [65], and ultimately promoting both their academic and life adjustment.

**Table 5:** Tests the mediating effects of teacher support and environment support on school adjustment of rural left-behind children respectively.

	Model 13	Model 14	Model 15	Model 16	Model 17	Model 18	Model 19	Model 20	Model 21	Model 22	Model 23	Model 24
	Academic adjustment	Academic adjustment	Life adjustment	Life adjustment	Students’ educational expectation	Students’ educational expectation	Students’ mental health	Students’ mental health	Academic adjustment	Academic adjustment	Life adjustment	Life adjustment
Teacher support												
Teachers’ academic support	0.103***	0.087***	0.100 *	0.079	0.154***	0.157***	0.016	0.019	0.078***	0.065**	0.103	0.086
Teachers’ emotional support	0.056***	0.043**	0.319***	0.297***	0.066 *	0.059	0.176***	0.139***	0.036 *	0.029	0.232***	0.230***
Teachers’ relationship support	0.134***	0.107***	0.484***	0.443***	0.140***	0.123***	0.264***	0.191***	0.116***	0.090***	0.354***	0.347***
Students’ educational expectation									0.109***	0.099***	0.162***	0.152***
Students’ mental health									0.057***	0.058***	0.328***	0.314***
Adjusted R^2^	0.091	0.132	0.182	0.219	0.039	0.053	0.064	0.112	0.142	0.171	0.265	0.287
	Model 25	Model 26	Model 27	Model 28	Model 29	Model 30	Model 31	Model 32	Model 33	Model 34	Model 35	Model 36
	Academic adjustment	Academic adjustment	Life adjustment	Life adjustment	Students’ educational expectation	Students’ educational expectation	Students’ mental health	Students’ mental health	Academic adjustment	Academic adjustment	Life adjustment	Life adjustment
Environment support												
School soft environment support	0.062***	0.040***	0.169***	0.140***	−0.039	−0.071**	0.089***	0.069**	0.067***	0.047***	0.170***	0.151***
School hard environment support	0.040***	0.032**	0.069**	0.057**	0.014	0.007	0.043 *	0.038	0.032***	0.026**	0.056**	0.051 *
Students’ educational expectation									0.139***	0.122***	0.251***	0.231***
Students’ mental health									0.073***	0.064***	0.385***	0.349***
Adjusted R^2^	0.02	0.094	0.022	0.104	0.000	0.023	0.008	0.074	0.113	0.157	0.188	0.221

Note: (1) All of even-numbered models all control grade, gender, parental relationship, parent-child relationship, parents’ education level, only child, and family economic status. (2) Among the control variables, seventh grade, girls, only children, and difficulties are used as the benchmark group. (3) * , **, and *** indicate significance at the 10%, 5%, and 1% significance levels respectively.

### 5.2 Comparison of the impact on left-behind children and non-left-behind children

Table 6 reports the results of teacher support and environment support affecting rural non-left-behind children’s school adjustment. As shown in Model 37 to Model 40, teachers’ emotional support and teachers’ relationship support have significant positive effects on rural non-left-behind children’s school adjustment and life adjustment, and teachers’ academic support has no significant effect on rural non-left-behind children’s school adjustment and life adjustment. As shown in Model 41 and Model 42, teachers’ emotional support and teachers’ relationship support significantly increase the educational expectation of rural non-left-behind children (The effect of teacher emotional support on enhancing educational expectations for rural non-left-behind children is not significant), while teachers’ academic support has no significant effect on students’ educational expectation. As shown in Model 43 and Model 44, teachers’ emotional support and teachers’ relationship support also significantly improve students’ mental health, and teachers’ academic support has no significant effect on students’ mental health. Model 45 to Model 48 verified that teachers’ emotional support and teachers’ relationship support improved rural non-left-behind children’s academic adjustment and life adjustment by improving students’ educational expectation and mental health, respectively, and teachers’ relationship support significantly improved rural non-left-behind children’s school adjustment ability and had a significant mediating effect through mental health.

**Table 6:** Tests the mediating effects of teacher support and environment support on school adjustment of rural non-left-behind children respectively.

	Model 37	Model 38	Model 39	Model 40	Model 41	Model 42	Model 43	Model 44	Model 45	Model 46	Model 47	Model 48
	Academic adjustment	Academic adjustment	Life adjustment	Life adjustment	Students’ educational expectation	Students’ educational expectation	Students’ mental health	Students’ mental health	Academic adjustment	Academic adjustment	Life adjustment	Life adjustment
Teacher support												
Teachers’ academic support	0.008	−0.003	0.093	0.086	0.021	0.053	0.108	0.039	−0.004	−0.006	0.097	0.108
Teachers’ emotional support	0.155***	0.130***	0.352***	0.281***	0.107**	0.051	0.136***	0.089 *	0.131***	0.117***	0.298***	0.254***
Teachers’ relationship support	0.148***	0.094***	0.377***	0.366***	0.234***	0.117***	0.197***	0.157***	0.105***	0.070***	0.289***	0.302***
Students’ educational expectation									0.127***	0.098***	0.160***	0.129***
Students’ mental health									0.065***	0.066***	0.246***	0.235***
Adjusted R2	0.112	0.144	0.135	0.180	0.045	0.149	0.038	0.106	0.172	0.184	0.205	0.239
	Model 49	Model 50	Model 51	Model 52	Model 53	Model 54	Model 55	Model 56	Model 57	Model 58	Model 59	Model 60
	Academic adjustment	Academic adjustment	Life adjustment	Life adjustment	Students’ educational expectation	Students’ educational expectation	Students’ mental health	Students’ mental health	Academic adjustment	Academic adjustment	Life adjustment	Life adjustment
Environment support												
School soft environment support	0.062***	0.024	0.228***	0.175***	0.050	−0.049	0.136***	0.109**	0.048**	0.024	0.171***	0.151***
School hard environment support	0.034***	0.021	−0.004	−0.013	0.037	−0.002	−0.026	−0.015	0.039***	0.032**	−0.002	−0.008
Students’ educational expectation									0.161***	0.120***	0.249***	0.188***
Students’ mental health									0.077***	0.069***	0.292***	0.265***
Adjusted R^2^	0.014	0.103	0.018	0.100	0.003	0.155	0.006	0.089	0.125	0.156	0.131	0.181

Note: (1) All of even-numbered models control grade, gender, parental relationship, parent-child relationship, parents’ education level, only child, and family economic status. (2) Among the control variables, seventh grade, girls, only children, and difficulties are used as the benchmark group. (3) * , **, and *** indicate significant significance at 10%, 5%, and 1% significance levels respectively.

Teacher academic support only affects the academic adjustment of rural left-behind children, while having no impact on the academic and life adjustment of rural non-left-behind children. Teacher emotional support only influences the life adjustment of rural left-behind children, but significantly affects both academic and life adjustment for rural non-left-behind children. Teacher relationship support has a significant effect on the academic and life adjustment of both groups, reflecting the heterogeneity in the impact of different types of teacher support on student populations. This may be because rural children living with their parents receive academic supervision and can communicate face-to-face with their parents, who help alleviate academic and psychological pressures, thereby meeting their learning and emotional needs, which substitutes for teacher’s academic and emotional support. However, teacher emotional support appears to have a more pronounced positive effect on the mental health of rural left-behind children. This suggests that the lack of parental presence and emotional support for left-behind children makes teacher emotional support a crucial compensatory factor. Furthermore, it highlights the unique value of parent-child education in stable family environments, which cannot be replaced by other forms of support like teacher’s academic assistance. The stable qualities of teacher relationship support, such as responsibility and patience, reflect attributes unique to teachers and demonstrate their unique value in promoting student development, which parents cannot replicate.

From the perspective of environmental support, the soft environmental support within schools has a significant positive impact on the life adjustment of both rural left-behind and non-left-behind children, influencing their life adjustment through the intermediary effect of improved psychological health. Meanwhile, hard environment support also positively affects the academic adjustment of both groups, though its impact on life adjustment is less pronounced (with hard environmental support still significantly benefiting the life adjustment of rural left-behind children). This suggests that increased government investment in rural school infrastructure and improved educational conditions provide strong support for rural students’ learning. However, more importantly, rural schools need to enhance management levels and adopt intrinsic development strategies to improve educational quality, thereby laying a solid foundation for the development of rural children.

### 5.3 Comprehensive discussion

School adjustment is crucial for students’ future educational achievement and career development. Students who adjust well exhibit stronger academic motivation and better relationships with teachers and peers, which not only enhances their academic performance but also fosters positive values and prosocial behaviors. Due to the lack of opportunities for reunion with their parents, the parent-child educational function in family education is difficult to realize. The lack of opportunities for family reunification hampers parental education, leading to significant psychological and behavioral differences between left-behind and non-left-behind children. Strong teacher support and environment support can improve students’ school adjustment, partially mitigating the negative impact caused by the dysfunction of family education on left-behind rural children.

The influence of teacher support is multidimensional and complex. A teacher’s charisma can have a profound impact on students. Responsibility and patience are fundamental qualities for teachers. Some rural left-behind children may have fragile mental health and struggle to communicate proactively with teachers, which can lead to isolation and persistent psychological issues. The most direct way to implement care policies for left-behind children is for teachers to assume responsibility for support, encouraging teachers to patiently communicate with these children. By helping left-behind children address issues arising from parental absence, teachers can build trust with them and provide a sense of care, which can reverse their unhealthy psychological patterns, instill their confidence about the future, and raise their educational expectations, ultimately motivating their academic engagement. For left-behind children, teacher support, both academic and emotional, compensates for the lack of parental guidance, while teacher-student relationships positively impact all students, fulfilling a unique supportive role.

Additionally, the school environment can also provide positive support. A school’s soft environment reflects the quality of its management. Organized management can cultivate a constructive campus culture. Individuals within such an environment are influenced by this atmosphere, which can encourage left-behind children to overcome their isolation, become more accepting of themselves, and gradually develop a mindset that aligns with their surroundings. This underscores the inherent value of the environment in fostering personal growth.

In summary, the mechanisms by which school support affects the school adjustment of rural left-behind children are complex. This includes not only the influence of teacher support but also the impact of environmental support. Moreover, the different dimensions of teacher and environmental support can lead to varied effects. The underlying mechanisms of these differentiated impacts may also vary significantly. Additionally, when considering group differences, the interactions among teacher support, the group, and the environment further complicate these influences.

## 6 Summary

This paper uses data from the China Education Panel Survey (CEPS) in 2013–2014 to explore the effects of school support on rural left-behind children’s school adjustment and the mechanisms of its occurrence based on linear regression and structural equation model, and obtains the following main conclusions: firstly, school support can effectively improve rural left-behind children’s school adjustment ability; secondly, school support improves students’ school adjustment ability by improving their educational expectation and mental health, especially the influence effects of teachers’ relationship support and school soft environment support in school support are significant; thirdly, teacher support has a greater effect on students’ school adjustment ability than environment support, and soft environment support enhances students’ school adjustment ability by improving their mental health, while the impact of hard environment support is weaker.

## 7 Significance of the study

The issue of school adjustment for left-behind children in rural areas relates directly to their quality of learning and life at school, as well as their future educational achievement and career development. Addressing how to implement effective interventions for these children is a crucial consideration in both policy and practice. This paper suggests that both teachers and the environment play significant roles in improving school adjustment of left-behind children. Multidimensional support from teachers in terms of academics, emotions, and relationships can enhance these children’s educational expectations and mental health, ultimately improving their school adjustment abilities and providing theoretical support for the design of intervention policies and implementation pathways.

Firstly, the protective role of teacher support in the school adjustment of left-behind children must be maximized. Academic support from teachers, such as tutoring, can compensate for the lack of parental academic supervision, thereby enhancing educational expectations and improving their adjustment to learning. Secondly, teachers should also prioritize emotional communication with left-behind children, addressing their psychological concerns in a timely manner to maintain their mental health and strengthen their school adjustment. More importantly, it is essential to inspire a sense of professional identity among rural teachers; thus, it is necessary to continue improving the training system for primary and secondary school teachers to enhance their professional skills and ethical standards.

Additionally, this study provides evidence for understanding the relationship between school education and family education. Both teachers and parents are “significant others” in a child’s development; whether in school or at home, each plays a unique role in education. For instance, the absence of academic support at home can be supplemented by teachers’ academic assistance. The unique value of schools and teachers in students’ growth is irreplaceable, as high-quality relationships between teachers and students, as well as among peers, are essential for personal development. Furthermore, establishing a communication and coordination mechanism between home and school to enhance the teacher-parent relationship can maximize the developmental rights of disadvantaged students.

## 8 Research limitations and future directions

While this study provides evidence that school support improves the school adjustment of left-behind children in rural areas through the mediating paths of educational expectations and mental health, it also has some limitations. Firstly, the use of cross-sectional data from 2013-2014 diminishes the persuasiveness of the conclusions, as such data only captures the momentary state of concepts like teacher support and school adjustment, failing to elucidate their underlying causal relationships. Secondly, the mechanisms by which school support influences the school adjustment of left-behind children remain unclear, particularly regarding the impact of the physical school environment.

To further explore the relationship between teacher support and environmental support, this study finds a significant correlation between the two. Future research can expand on this relationship from a person-environment interaction perspective to uncover richer meanings of school support. Additionally, conducting longitudinal studies on left-behind children could provide a dynamic view of how school support affects their adjustment over time. Furthermore, exploring other mediating pathways through which school support impacts these children could broaden the theoretical understanding of such studies.
